# Effects of type 1 diabetes mellitus on lumbar disc degeneration: a retrospective study of 118 patients

**DOI:** 10.1186/s13018-020-01784-6

**Published:** 2020-07-25

**Authors:** Rui Chen, Xinjie Liang, Tianji Huang, Weiyang Zhong, Xiaoji Luo

**Affiliations:** 1 Department of Neurology, The First Affiliated Hospital of Chongqing Medical University, Chongqing, 400016 P. R. China; 2 Department of Pain Management, The First Affiliated Hospital of Chongqing Medical University, Chongqing, 400016 P. R. China; 3 Department of Orthopedic Surgery, The First Affiliated Hospital of Chongqing Medical University, Chongqing, 400016 P. R. China

**Keywords:** Type 1 diabetes, Lumbar disc degeneration, Risk factor

## Abstract

**Background:**

The study aimed to investigate the correlation between type 1 diabetes (T1D) and lumbar disc degeneration (LDD).

**Methods:**

A retrospective analysis of 118 patients with T1D recruited from January 2014 to March 2019 was performed, and multivariate logistic regression was used to analyse the incidence of T1D; the age, sex, and body mass index (BMI) of the patients; the disease duration and the glycosylated haemoglobin and venous blood glucose levels. All patients who suffered low back pain were assessed by MRI using the Pfirrmann grading system.

**Results:**

A total of 118 patients with an average age of 36.99 ± 17.01 (8–85 years) were reviewed. The mean hospitalization duration, venous glucose fluctuation range, glycated haemoglobin level, highest venous glucose level, venous glucose level, and disease course duration were 13.98 ± 10.16 days, 14.99 ± 5.87 mmol/L, 9.85 ± 2.52 mmol/L, 25.29 ± 7.92 mmol/L, 13.03 ± 5.75 mmol/L and 7.30 ± 8.41 years. The average Pfirrmann scores of the different discs were 2.20 ± 0.62 (L1–2), 2.35 ± 0.67 (L2–3), 2.90 ± 0.45 (L3–4), 4.20 ± 0.52 (L4–5) and 4.10 ± 0.72 (L5–S1). The patients with T1D showed severe disc degeneration. The male sex, glycosylated haemoglobin, venous glucose and venous glucose fluctuations were significantly associated with LDD (*P* < 0.05).

**Conclusions:**

Glycosylated haemoglobin, the male, venous glucose and the venous glucose fluctuation range were risk factors for LDD.

## Background

Diabetes mellitus (DM) is a common disease with a worldwide incidence rate of approximately 8%. There are 1.14 million diabetic patients, and the annual rate of increase is 1.2 million; 5% of these patients have type 1 diabetes (T1D) [1–5]. T1D is an autoimmune disease in which beta cells that produce insulin are destroyed. Furthermore, because of the early onset, T1D can affect many organs, including the bone and cartilage, and these changes result in catabolic and anabolic unbalanced responses that lead to intervertebral disc degeneration [6–8]. Hyperglycaemia can cause metabolic disorders of carbohydrates, proteins and other substances, as well as microvascular disease, resulting in insufficient nutrient supply to the intervertebral discs. Furthermore, T1D may contribute to intervertebral disc degeneration by promoting aggrecan degradation and apoptosis. LDD is a major cause of low back pain and leg pain, which can cause health problems for individuals and affect their daily life and work [9, 10].

Previous studies have focused on the relationship between type 2 DM and LDD and concluded that DM is a risk factor for LDD, and that DM is related to spinal stenosis [6–10]. Due to T1D being an immune disease with an early onset time and glucose control being difficult, T1D can result in early degeneration of the intervertebral disc (IVD). In our study, we investigated the relationship between T1D and LDD assessed by the Pfirrmann scoring system with MRI.

## Materials and methods

### Study design

This study was approved by the Institutional Review Board of the First Affiliated Hospital of Chongqing Medical University and conducted according to the principles of the Declaration of Helsinki. All the patients provided written informed consent to participate in our study prior to the storage of their data in the hospital database.

Patients recruited from January 2014 to March 2019 were reviewed retrospectively. The inclusion criteria were adults with (1) symptoms of diabetes and diabetic ketosis that occurred within 6 months of the onset of diabetes, (2) insulin-dependent therapy and (3) fasting C peptide (FCP) and postprandial C peptide (PCP) levels of ≤ 200 pmol/L and ≤ 400 pmol/L, respectively. The exclusion criteria were patients with type 2 DM, gestational diabetes, fulminant type 1 diabetes, adult occult autoimmune diabetes, adolescent adult diabetes, mitochondrial diabetes, or other special types of diabetes and patients suffering from serious non-diabetic related heart, liver, kidney or other organ-related diseases.

### Outcome assessments

LDD grading was performed independently by an experienced spine surgeon with standard T2-weighted turbo spin-echo sagittal images and the five-level Pfirrmann grading system [11].

### Statistical analysis

The statistical analysis was performed using the Statistical Analysis Software (SAS Institute Inc., Cary, NC, USA). Quantitative variables were described as the mean ± SD. Regression analysis was used to identify the relationship between T1D and LDD. Differences with a *P* value of < 0.05 were considered significant.

## Results

A total of 118 patients with an average age of 36.99 ± 17.01(8-85 years) were reviewed (Table 1). The mean hospitalization duration, venous glucose fluctuation range, glycated haemoglobin level, highest venous glucose level, venous glucose level and disease course duration which was 13.98 ± 10.16 days, 14.99 ± 5.87 mmol/L, 9.85 ± 2.52 mmol/L, 25.29 ± 7.92 mmol/L, 13.03 ± 5.75 mmol/L and 7.30 ± 8.41 years. The average Pfirrmann scores of the different discs were 2.20 ± 0.62 (L1–2), 2.35 ± 0.67 (L2–3), 2.90 ± 0.45 (L3–4), 4.20 ± 0.52 (L4–5) and 4.10 ± 0.72 (L5–S1) (Table 1).

**Table 1:** Demographic data

Index	Values	
Age	36.99 ± 17.01 (8–85)	
Male/female (*n*)	61/57	
Hospitalization duration (d)	13.98 ± 10.16	
Venous glucose fluctuation range (mmol/L)	14.99 ± 5.87	
Glycated haemoglobin (mmol/L)	9.85 ± 2.52	
Highest venous glucose (mmol/L)	25.29 ± 7.92	
Venous glucose (mmol/L)	13.03 ± 5.75	
Disease course (y) average	7.30 ± 8.41	
Pfirrmann scores		
L1–2	2.20 ± 0.62	
L2–3	2.35 ± 0.67	
L3–4	2.90 ± 0.45	
L4–5	4.20 ± 0.52	
L5–S1	4.10 ± 0.72	

**Fig. 1:**
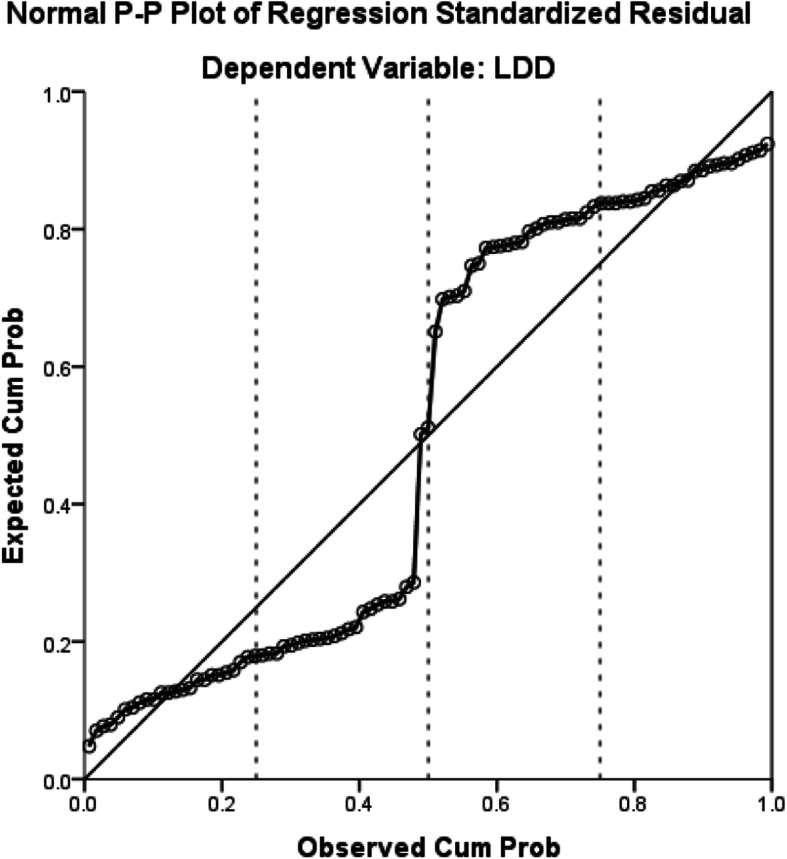
The relationship between venous glucose and LDD. Cum Prob, cumulative probability

**Fig. 2:**
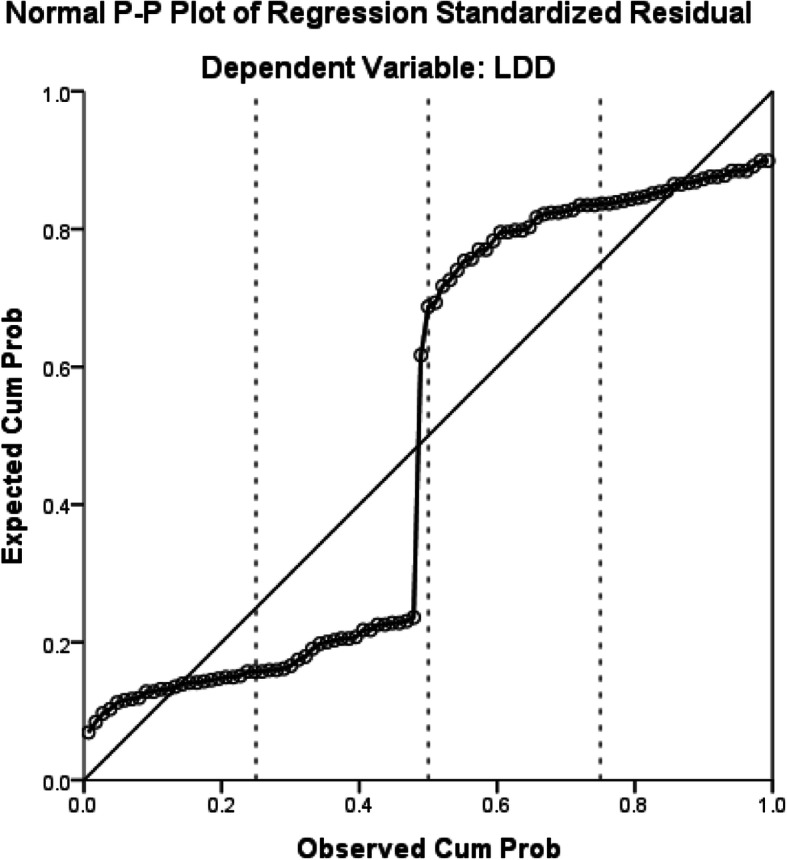
The relationship between glycated haemoglobin and LDD

**Fig. 3:**
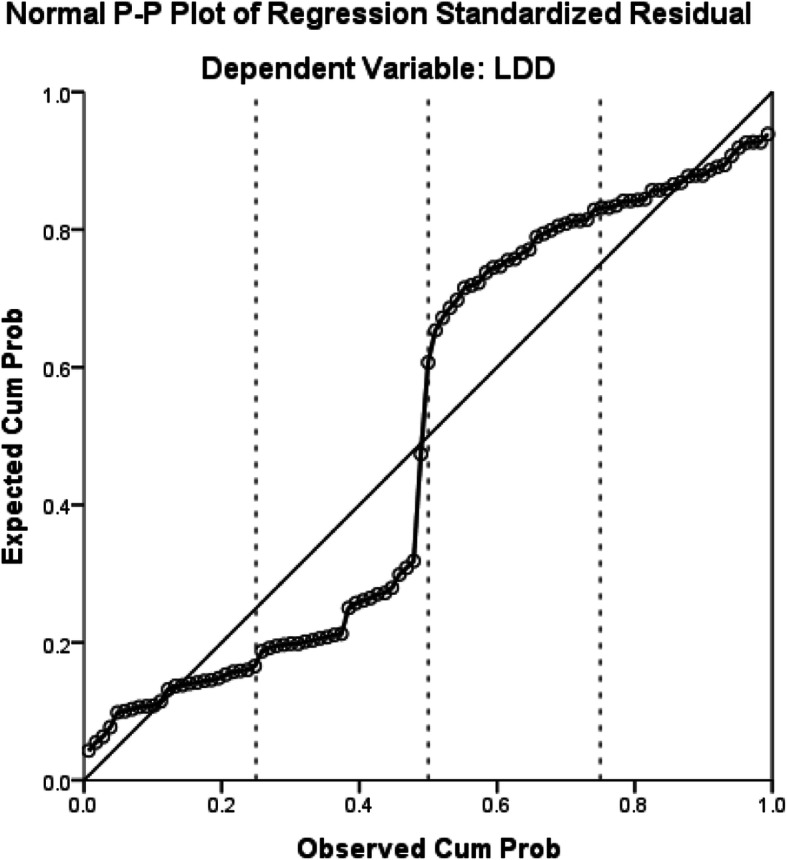
The relationship between venous glucose fluctuation and LDD

**Fig. 4:**
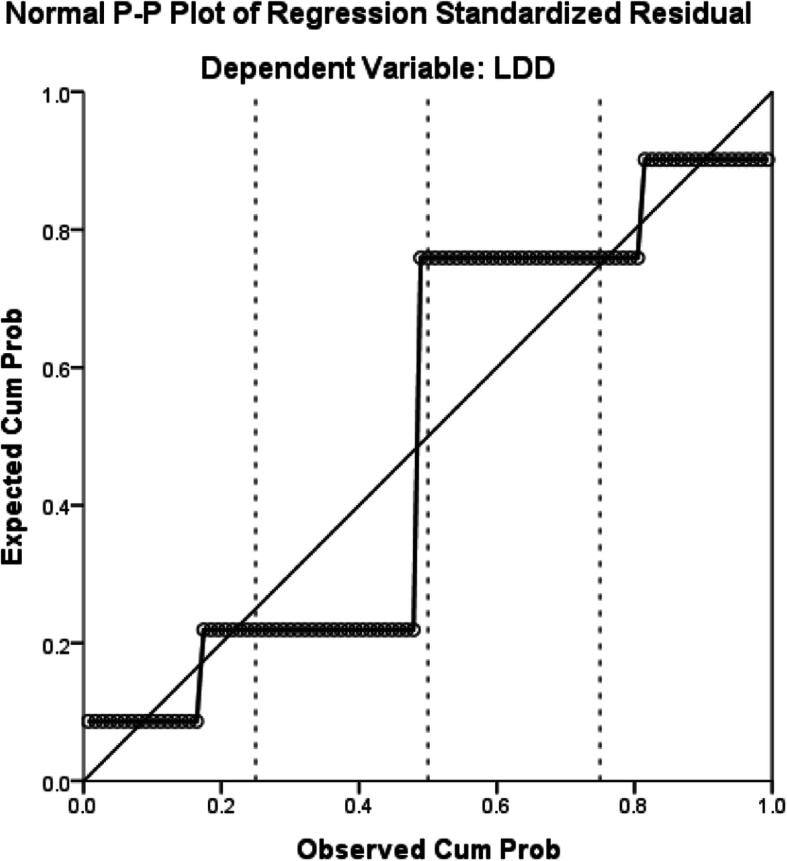
The relationship between sex and LDD

**Fig. 5:**
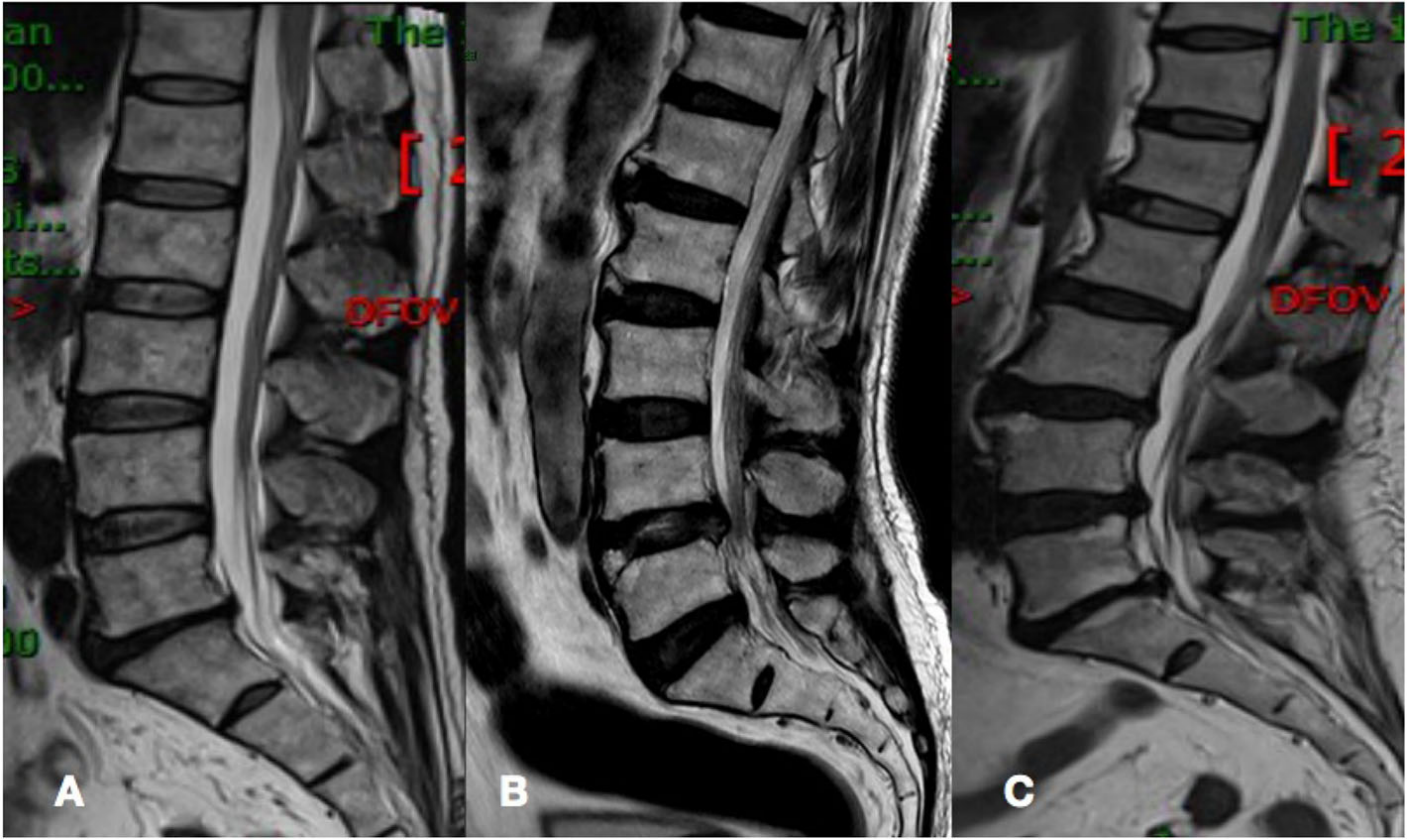
**a** A 30-year-old female patient with T1D for 6 years suffering low back pain for 5 years. The Pfirrmann scores from L1/2 to L4/5 are all 3, and L5/S1 are 4. **b** a 44-year-old male patient with T1D for 10 years suffering low back pain for 8 years. The Pfirrmann scores from L1/2 to L3/4 are all 4, and from L4/5 to L5/S1 are 5. **c** a 57-year-old female patient with T1D for 16 years suffering low back pain for 15 years. The Pfirrmann scores of L1/2 are 3 and from to L2/3 to L3/4 are all 4 and from L4/5 to L5/S1 are 5

The male sex, glycosylated haemoglobin, venous glucose and venous glucose fluctuations were significantly associated with LDD (*P* < 0.05) (Table 2). Regression analysis indicated that these factors have a linear relationship with LDD (Figs. 1, 2, 3, 4 and 5).

**Table 2:** Regression analysis between T1D and LDD

Index	*B* value	Standard value	Wald	OR	95%confidence interval	*P* value
Age	0.042	0.020	4.273	1.043	1.002–1.085	0.061
Male sex	1.232	0.583	4.469	3.428	1.094–1.743	0.035
Venous glucose fluctuation range (mmol/L)	0.120	0.056	4.537	1.127	1.010–1.259	0.033
Glycated haemoglobin (mmol/L)	0.300	0.131	5.247	1.350	1.044–1.745	0.022
Highest venous glucose (mmol/L)	− 0.026	0.034	0.576	0.974	0.911–1.042	0.448
Venous glucose (mmol/L)	0.146	0.053	7.674	1.157	1.044–1.283	0.036
BMI	0.184	0.125	2.162	1.202	0.952–1.537	0.141

## Discussion

T1D is considered an autoimmune disease that affects individuals from a young age and requires insulin-dependent treatment due to defects in insulin secretion in the individual. It has a tendency to ketoacidosis. Long-term chronic hyperglycaemia in individuals with T1D is associated with chronic complications, which are a threat to children and adolescents [12, 13]. Fifty percent of patients with T1D are adults, which has a large impact on social productivity and medical expenditures. T1D patients have relatively complicated treatment plans, a long course of disease and poor blood glucose control. According to the US Wisconsin Diabetes Registration Counseling Study data, only 1% of patients have high glycosylated haemoglobin (HbA1c) levels, while < 7% of the T1D patients in China have high HbA1c levels, leading to more challenge s[14, 15]. Furthermore, in China, the clinical features are more atypical, and the risk of complications associated with diabetes is much higher, which should be addressed.

LDD can cause low back pain, affect patients’ quality of life and increase medical expenses. Hence, it is very important to investigate the risk factors for LDD to prevent or delay its onset or progression. This study was the first to evaluate the association between T1D and LDD [4, 7, 10]. In previous studies, Hao et al., Mysliwiec LW et al. and Milette PC et al. [12–14] have found that the patients aged 40  ±  4.6 years had slight LDD in MRI, and 63.9% of the patients had 1.63 ± 0.68 of Pfirrmann scores while the average of Pfirrmann scores was 3.15 ± 0.95 in our study. One study reported that patients with type 2 diabetes (T2D) had more tendency to develop more severe LDD than those without T2D. The T2D duration had a positive correlation with severity of LDD which mean the patients with a worse control of T2D showed more severe LDD than those with a good control [10].

In our study, the male sex, glycosylated haemoglobin, venous glucose and venous glucose fluctuations were significantly associated with LDD. The incidence of T1D in male patients was higher than that in female patients, and the male patients had poorer control of glucose. Of the patients with T1D, the patients with poor control of glucose had more severe disc degeneration. This result showed that T1D was a risk factor for LDD. It has been reported that DM patients have a poorer outcome following lumbar discectomy than patients without DM, and that high preoperative glycated haemoglobin levels and long-term DM are risk factors for poor cervical laminoplasty outcomes in patients with T2D and cervical spondylotic myelopathy [10].

Now, we can conclude that there is a positive relationship between T1D and LDD. However, the underlying mechanisms remain unclear. Previous studies have suggested that in a hyperglycaemic environment, nucleus pulposus (NP) cells stop producing or even begin to degrade proteoglycan and collagen II, resulting in a decreased intravertebral pressure and a disruption in the structural integrity of the nucleus, and that hyperglycaemia enhances the formation of advanced glycation end products (AGEs) in the NP, which also leads to the progression of disc degeneration. Some studies have found that autophagy in diabetic rats is significantly higher than that in control rats, and it is believed that autophagy caused by hyperglycaemia can lead to degeneration in intervertebral disc cells [15–20]. Moreover, the microvessels of the vertebral endplates of diabetic animals are significantly narrowed, resulting in a decrease in blood supply and nutrition in the intervertebral disc. The vertebral endplate may become harder, further impairing its permeability to nutrients. This type of sclerosis is caused by the anabolic effects of DM on osteochondral metabolism [21–24]. Also, other studies found that high serum cholesterol and triglyceride levels are risk factors for atherosclerosis, which may be the cause of reduced blood supply to already poor vascularized IVD, and more understanding about IVD degeneration process was enhanced [25].

In our study, we also found that the average Pfirrmann scores in patients with poor control of DM indicated severe degeneration. For most patients with T1D, although good control of glucose is difficult, continuous adequate insulin therapy is essential. Not only is it able to maintain patients’ glucose levels in the normal range, but it is also able to avoid acute or chronic complications. Furthermore, after insulin treatment for the T1D patients, we must know that IVD is a complex pathological process. Conservative treatments, such as lifestyle changes, physical therapy, painkillers and rehabilitation, often fail. Except surgical treatments, the biotherapy requires more attention and understanding as its goal is to prevent and manage IVD degeneration. However, clinical applications of biotherapy are still have a long way, and more animal and clinical studies are needed to elucidate the role of mesenchymal stem cells and the role of gene therapy in the prevention and treatment of IVD degeneration [26].

To our knowledge, this was the first study to investigate the relationship between T1D and LDH with MRI. However, we want to declare that there are several limitations in this study. First, all included patients in our study were experiencing low back pain. Second, we did not conduct basic research on how T1D affects LDH. Hence, in the future, basic research studies on genes related to degeneration in intervertebral discs should be performed.

## Conclusion

Glycosylated haemoglobin, the male, venous glucose and venous glucose fluctuation range were risk factors for LDD.

## Data Availability

The datasets generated and/or analysed during the current study are not publicly available due to the data is confidential patient data but are available from the corresponding author on reasonable request.

## Abbreviations

T1D: Type 1 diabetes
LDD: Lumbar disc degeneration
BMI: Body mass index
DM: Diabetes mellitus
IVD: Intervertebral disc
FCP: Fasting C peptide
PCP: Postprandial C peptide
BP: Nucleus pulposus
AGEs: Advanced glycation end products
T2D: Type 2 diabetes
